# Rapid targeted somatic mutation analysis of solid tumors in routine clinical diagnostics

**DOI:** 10.18632/oncotarget.5190

**Published:** 2015-09-02

**Authors:** Gilda Magliacane, Greta Grassini, Paola Bartocci, Ilaria Francaviglia, Elena Dal Cin, Gianluca Barbieri, Gianluigi Arrigoni, Lorenza Pecciarini, Claudio Doglioni, Maria Giulia Cangi

**Affiliations:** ^1^ Unit of Pathology, IRCCS San Raffaele Scientific Institute, Milano, Italy; ^2^ Diatech Pharmacogenetics Company, Jesi, Italy

**Keywords:** Pathology, molecular diagnostics, solid tumors, high throughput mass-spectrometry, target therapy, mutations

## Abstract

Tumor genotyping is an essential step in routine clinical practice and pathology laboratories face a major challenge in being able to provide rapid, sensitive and updated molecular tests.

We developed a novel mass spectrometry multiplexed genotyping platform named PentaPanel to concurrently assess single nucleotide polymorphisms in 56 hotspots of the 5 most clinically relevant cancer genes, *KRAS, NRAS, BRAF, EGFR* and *PIK3CA* for a total of 221 detectable mutations. To both evaluate and validate the PentaPanel performance,we investigated 1025 tumor specimens of 6 different cancer types (carcinomas of colon, lung, breast, pancreas, and biliary tract, and melanomas), systematically addressing sensitivity, specificity, and reproducibility of our platform. Sanger sequencing was also performed for all the study samples.

Our data showed that PentaPanel is a high throughput and robust tool, allowing genotyping for targeted therapy selection of 10 patients in the same run, with a practical turnaround time of 2 working days. Importantly, it was successfully used to interrogate different DNAs isolated from routinely processed specimens (formalin-fixed paraffin embedded, frozen, and cytological samples), covering all the requirements of clinical tests.

In conclusion, the PentaPanel platform can provide an immediate, accurate and cost effective multiplex approach for clinically relevant gene mutation analysis in many solid tumors and its utility across many diseases can be particularly relevant in multiple clinical trials, including the new basket trial approach, aiming to identify appropriate targeted drug combination strategies.

## INTRODUCTION

The introduction of personalized therapy transformed the care of selected cancer patients: detection of critical cancer gene somatic mutations in clinical tumor samples better defines patient diagnosis, prognosis and more importantly indicates highly efficient targeted therapies with both health and economic benefits [1].

According to the approved antineoplastic targeted drugs, the mutational status of *EGFR, KRAS, NRAS, BRAF, PIK3CA* genes is routinely requested by the oncologist for the clinical management of patients with non-small cell lung carcinomas (NSCLC) [2–4], colorectal carcinomas (CRC) [5, 6], melanomas [7], breast carcinomas [8, 9]. In particular EGFR inhibitors for *EGFR*-mutant NSCLC and the mutation-selective RAF and MEK inhibitors for *BRAF*-mutant melanoma [10] are examples of successfully targeted therapy selection. Further, the presence of mutations in *RAS* family oncogenes is associated with a lack of response to targeted therapy: lung and colorectal carcinomas characterized by *KRAS* mutations and *KRAS* and *NRAS* mutations, respectively are unresponsive to treatment with anti-EGFR agents [6, 11].

The routine clinical testing of such alterations faces several challenges. First, routine tumor specimens are usually formalin-fixed and paraffin-embedded (FFPE) and formalin fixation effects are major problems in molecular diagnostics. Formalin induces chemical cross-links to proteins, RNA, and DNA molecules, with concomitant fragmentation of DNA [12, 13] and random nucleotide base changes, which can lead to false-positive results [14]. For these reasons, clinical mutation detection PCR-based assays should include two independent amplifications of DNA extracted from FFPE samples in order to ensure accurate results, as suggested by molecular testing guidelines [15, 16].

Second, when offering optimal patient care, applied technologies should allow fast implementation of available assays in order to respond to new targets development, clinical trials testing, and new rules imposed by drug control agencies. As recently shown in metastatic colorectal cancer treatment, 17% of the *KRAS* exon 2 wild type patients, who do not respond to anti-EGFR treatment, harbor mutations in *KRAS* exon 3 and 4 and *NRAS* exon 2, 3 and 4 [6]. This evidence was rapidly followed by a directive of the European Medicines Agency (EMA) that restricted the use of panitumumab (Vectibix^®^) and cetuximab (Erbitux^®^) to patients with *KRAS* and *NRAS* (exon 2, 3 and 4) wild type metastatic colorectal cancer [17].

Third, an increasing need is determining the status of multiple clinically relevant genes in single sample-derived tumor. In fact, sequential testing using single gene analysis may be time consuming and need further invasive biopsy procedures, which could be avoided by testing for all informative markers in parallel when the first diagnosis is made [16, 18].

Herein we report the development and validation of a mass spectrometry multiplexed genotyping platform named PentaPanel, that comprises all the criteria defining a molecular diagnostic test: high performance in sensitivity, specificity, spectrum of detected mutations, and turnaround time.

Our approach is designed to concurrently detect 221 recurrent somatic point mutations in the 5 most relevant genes to solid tumors targeted therapies, *EGFR, KRAS, NRAS, BRAF*, and *PIK3CA*, applicable to 10 patients in the same run with a 2 day turnaround time.

Results of the PentaPanel genotyping application to the routine molecular pathology diagnostics of 1025 cases at our Institution are also reported.

## RESULTS

### PentaPanel platform design and characteristics: bidirectional analysis for detection of somatic mutations

**Figure 1:**
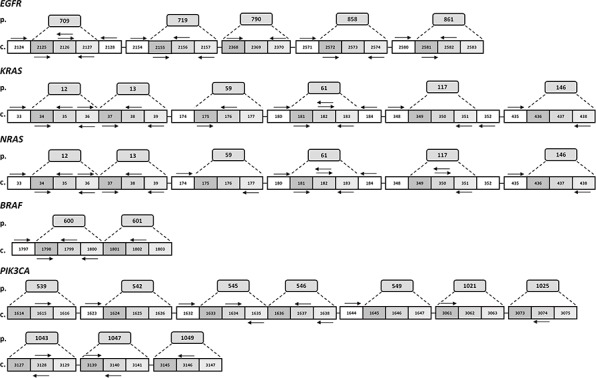
Schematic representation of PentaPanel platform Hotspot aminoacids (p.) and corresponding coding codons (c.) are schematically represented by boxes for each of the five genes, *EGFR, KRAS, NRAS, BRAF*, and *PIK3CA*. Arrows show extension primers adjacent to the targeted mutation sites. Most frequently mutated nucleotides are analyzed by bidirectional approach.

**Figure 2:**
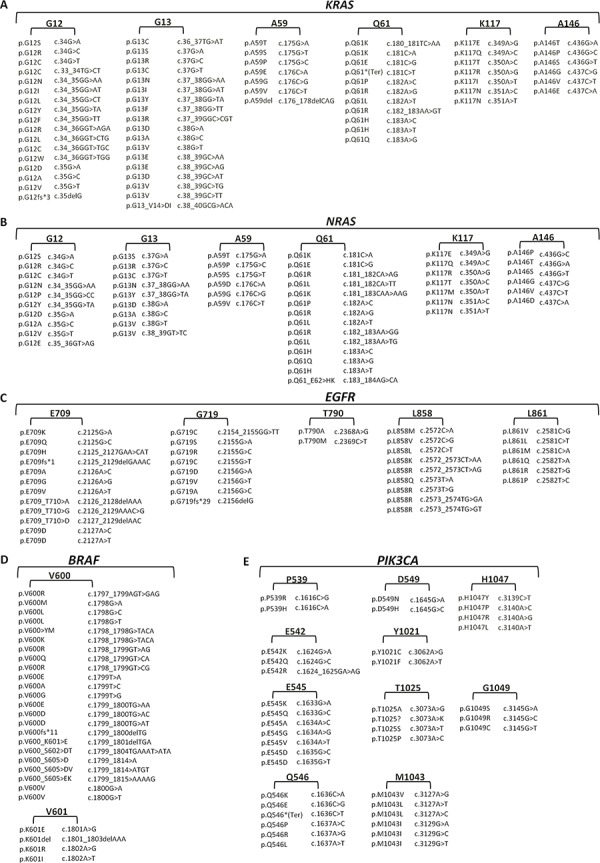
*KRAS, NRAS, BRAF, EGFR* and *PIK3CA* detectable mutations by PentaPanel platform Comprehensive list of the 221 mutations covered by PentaPanel in *KRAS* **A.** *NRAS* **B.** *EGFR* **C.** *BRAF* **D.** and *PIK3CA* **E.** genes; for each hotspot codon both amino acid and nucleotide changes are shown.

We successfully designed 80 multiplexed assays in order to analyze single nucleotide polymorphisms (SNPs) in 56 hotspots of *KRAS, NRAS, BRAF, EGFR* and *PIK3CA*. In particular for 24 sites of *EGFR, KRAS, NRAS*, and *BRAF*, which are mutated at high frequency as reported by the online Catalogue Of Somatic Mutations in Cancer (COSMIC), we included both duplicate amplifications, in separate wells, and bidirectional single base pair extensions (FIG. 1). Overall, the total 80 assays, variously combined in 8 wells depending on their extension product masses, can simultaneously detect the presence/absence of 221 mutations of *KRAS, NRAS, BRAF, EGFR* and *PIK3CA* (FIG. 2).

Notably, the PentaPanel allowed targeted therapy selection genotyping of 10 patients in the same run with a practical turnaround time of 2 working days, including data analysis, evaluation and reporting (7 hours first day-1 hour hands-on; 2 hours second day-15 min hands-on, 30–60 min for analysis and report generation).

**Figure 3:**
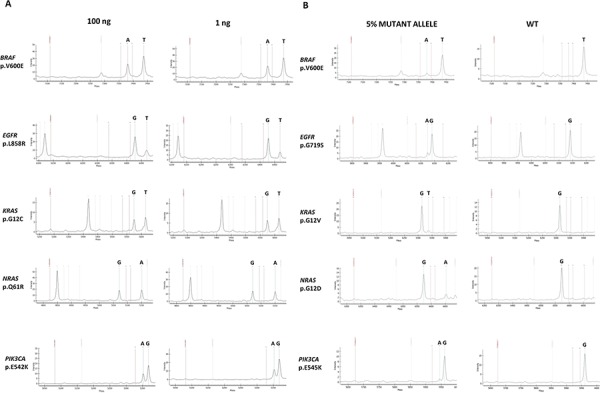
PentaPanel performance **A.** Representative spectra of samples with high (100 ng) or low (1ng) DNA template input showing similar frequency of the detected mutations; **B.** Representative spectra of the sensitivity study showing positive calls for mutations at 5% dilutions.

Our PentaPanel analysis was performed using 40ng (5ng/well) of genomic DNA template, although we showed that it is possible to get successful analysis using a minimum amount of 8 ng DNA (1ng/well). The minimum amount was based on validation studies performed on dilution series of FFPE-derived DNA with known mutations: mutation frequency of a sample with high (100 ng) DNA was equivalent to that with low (1ng) DNA (FIG. 3A).

To determine the analytical sensitivity of the platform we tested serial dilutions of genomic DNA extracted from FFPE samples with known mutant allele frequencies (20%, 10%, 5%, and 2.5%); we analyzed at least one representative mutation in each of the 8 wells: *BRAF* p.V600E, *KRAS* p.G12D, *KRAS* p.G12V, *NRAS* p.G12D, *NRAS* p.Q61K, *EGFR* p.G719S, *EGFR* p.T790M, *EGFR* p.L858R, *PIK3CA* p.E542K, *PIK3CA* p.E545K mutations. For *BRAF* p.V600E, *KRAS* p.G12D, *KRAS* p.G12V, *NRAS* p.Q61K, and *PIK3CA* p.E542K mutations commercially pre-designed reference standards were also utilized.

All the tested mutations were reproducibly (3 replicates) detected at a dilution of 5% (FIG. 3B). For *KRAS* p.G12V and *EGFR* p.G719S the detection limit was even lower, 2.5%. Same results were obtained using both FFPE-derived DNA and reference standards.

The reproducibility of the PentaPanel was also evaluated by comparing the results at the two independent laboratory sites and different users: the agreement was 100% for both mutation-positive and wild type specimens.

**Figure 4:**
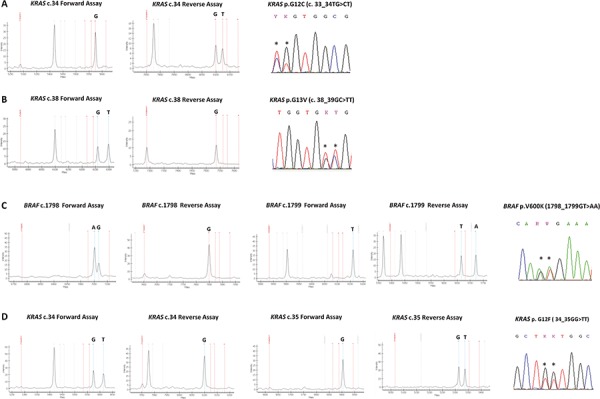
Spectra of bidirectional assays in representative complex mutations **A.** *KRAS* nucleotide c.34, forward assay is WT and reverse assay shows a mutation call (G > T); *KRAS* p.G12C (c.33_34TG > CT) mutation, as shown by Sanger sequencing. Mutation not yet listed in the COSMIC database **B.** *KRAS* nucleotide c.38, forward assay shows a mutation call (G > T) and reverse assay is WT; *KRAS* p.G13V (c.38_39GC > TT) mutation, as shown by Sanger sequencing **C.** *BRAF* nucleotide c.1798, forward assay shows a mutation call (G > A) and reverse assay is WT; *BRAF* nucleotide c.1799, forward assay is WT and reverse assay shows a mutation call (T > A); *BRAF* p.V600K (c.1798_1799GT > AA) mutation, as shown by Sanger sequencing. **D.** *KRAS* nucleotide c.34, forward assay shows a mutation call (G > T) and reverse assay is WT; *KRAS* nucleotide c.35, forward assay is WT and reverse assay shows a mutation call (G > T); *KRAS* p.G12F (c.34_35GG > TT) mutation, as shown by Sanger sequencing. By using a single assay approach all these mutations could be missed, with false negative results.

Importantly, for the first time our assays design allowed the bidirectional analysis of 24 highly frequently mutated sites, meaning that it comprised two separate assays assessing the presence/absence of the mutation in both the forward and the reverse strands. This strategy was critical for the detection of complex mutations such as dinucleotide substitutions occurring in both *BRAF* and *KRAS* genes: these mutations when tested by a unidirectional assay were missed as shown in FIG. 4. Moreover, by using the bidirectional approach, each result at a specific site was confirmed by two independent assays (in separated wells of the same run) as recommended by international guidelines [15, 16].

### PentaPanel platform performance and validation

For this study a total of 1025 clinical tumor specimens derived from 6 different cancer types, for which mutation analysis of selected genes had been requested as a part of clinical care, were analyzed using the PentaPanel platform. The samples included adenocarcinomas (*n* = 783) of the lung, colon, pancreas, and biliary tract, melanomas (*n* = 62), and breast carcinomas (*n* = 180). Of the total 1025 specimens, 910 derived from FFPE tissues, 73 were obtained from fresh frozen tissue and 42 were cytological stained smears. Tumor content exceeded 50% in all samples, as estimated by pathological review.

For PentaPanel platform validation, Sanger sequencing was performed for all the study specimens, as the common reference method for detecting somatic mutations in tumors [15]. In particular by Sanger sequencing we analyzed: *EGFR* (exons 18, 20, and 21) in lung adenocarcinomas and breast carcinomas; *KRAS* (exons 2, 3, and 4) in colon, lung, pancreas, biliary tract adenocarcinomas; *BRAF* (exon 15) in colon, lung, pancreas, biliary tract adenocarcinomas, and melanomas; *NRAS* (exons 2, 3, and 4) in colon adenocarcinomas, and melanomas; *PIK3CA* (exons 9 and 20) in colon adenocarcinomas and breast carcinomas. *EGFR* exon 19 deletions were analyzed by sizing assay in lung adenocarcinomas and breast carcinomas.

In five cases (2 lung adenocarcinomas, 1 breast carcinoma, and 2 pancreatic adenocarcinomas, all FFPE tissues), DNA quality was very poor and neither the PentaPanel platform nor the Sanger sequencing protocols were successful in obtaining PCR amplificates; 1020 specimens were then suitable for mutation analysis with an overall success rate of 99.5% (1020/1025) of the samples being profiled. This remarkably high genotype success rate is essentially attributable to the PentaPanel platform: in fact, the number of non-analyzable (NA) samples for Sanger sequencing was 28 (all FFPE tissues), with a success rate of 97.3% (997/1025). Overall, we observed a reduction in the number of NA samples, with 2% of samples rescued by PentaPanel approach versus conventional sequencing. Moreover, by PentaPanel we were able to successfully analyze all types of specimens, including cytological stained smears, as shown in Table 1, for which the low DNA yield is always a limiting factor.

**Table 1:** FFPE, fresh frozen, and cytological specimens successfully analyzed by PentaPanel

Cancer Type	Tested samples	FFPE samples	Fresh/Frozen samples	Cytologic samples	Mutated samples
**Colorectal**	386	346	40	−	245 (63%)
**Lung**	316	261	13	42	158 (50%)
**Melanoma**	62	55	7	−	44 (71%)
**Breast**	180	177	3	−	48 (26%)
**Pancreatic**	65	55	10	−	53 (81%)
**Biliary Tract**	11	11	−	−	4 (36%)
**Total**	1020	905	73	42	552

Concordance between PentaPanel platform and Sanger sequencing was determined using data from samples that were analyzed by both methods (i.e. we excluded the 23 samples that failed by Sanger sequencing). The overall percentage agreement between the two methods was 97.8% (975/997). In particular 22 samples, which were wild type for all the studied genes by Sanger sequencing, resulted mutated by PentaPanel: twenty were mutated in *KRAS* codon 12 and two in *BRAF* codon 600. The 22 negative cases for *KRAS* and *BRAF* mutations by standard sequencing were re-evaluated by the more sensitive LNA-PCR/Sequencing method: all the mutations detected by the PentaPanel were confirmed (concordance rate 100%) and no false positives were observed. Thus, we conclude that all the mutations could be detected by PentaPanel with a specificity of 100%.

### Frequency and distribution of somatic mutations

In our series of 1020 patients, a total of 605 somatic non-synonymous mutations in the 5 genes were found: 569 single nucleotide variations and 36 complex mutations were observed, as summarized in Table 2. Remarkably, among the total 30 *BRAF* mutations found in melanomas (30/62, 48%), we detected 6 complex mutations (1 p.V600E2, c.1799_1800TG > AA and 5 pV600K, c.1798_1799TG > AA), 20% (6/30) of the *BRAF* mutations, showing that the frequency of these events is not low.

**Table 2:** Single nucleotide and complex mutations identified in the current study

Amino acid	DNA	Cancer type			
		Lung	Colorectal	Melanoma	Others
***KRAS***		**108**	**197**	**0**	**57**
p.G12A	c.35G > C	10	9	−	1
p.G12C	c.34G > T	35	12	−	1
p.G12C	c.33_34TG > CT	−	2	−	−
p.G12D	c.35G > A	24	65	−	21
p.G12F	c.34_35GG > TT	3	1	−	−
p.G12H	c.34_35GG > CA	−	−	−	1
p.G12R	c.34G > C	1	1	−	5
p.G12S	c.34G > A	3	9	−	−
p.G12V	c.35G > T	18	42	0	23
p.G13C	c.37G > T	2	2	−	1
p.G13D	c.38G > A	1	24	−	−
p.G13R	c.37G > C	1	−	−	−
p.G13V	c.38G > T	1	1	−	−
p.A59T	c.175G > A	−	1	−	1
p.Q61H	c.183A > C	5	6	−	3
p.Q61L	c.182A > T	1	1	−	−
p.Q61P	c.182A > C	−	1	−	−
p.Q61K	c.180_181TC > AA	1	2	−	−
p.Q61R	c.182A > G	2	−	−	−
p.K117R	c.350 > G	−	1	−	−
p.K117N	c.351G > C	−	3	−	−
p.A146P	c.436G > C	−	3	−	−
p.A146T	c.436G > A	−	10	−	−
p.A146V	c.437C > T	−	1	−	−
***NRAS***		**0**	**14**	**14**	**0**
p.G12A	c.35G > C	−	−	1	−
p.G12C	c.34G > T	−	1	−	−
p.G12D	c.35G > A	−	6	−	−
p.G13R	c.37G > C	−	−	1	−
p.G13V	c.38G > T	−	1	−	−
p.Q61H	c.183A > C	−	1	−	−
p.Q61K	c.181C > A	−	3	4	−
p.Q61L	c.182A > T	−	1	4	−
p.Q61R	c.182A > G	−	1	4	−
***BRAF***		**5**	**22**	**30**	**1**
p.V600E	c.1799T > A	5	22	23	1
p.V600E2	c.1799_1800TG > AA	−	−	1	−
p.V600K	c.1798_1799GT > AA	−	−	5	−
p.K601E	c.1801A > G	−	−	1	−
***EGFR***		**41**	**0**	**0**	**0**
p. E709-T710del insA	c.2126_2128delAAA	1	−	−	−
p. E746-A750del	c.2232_2249 > AAA	14	−	−	−
p. E746-T751del insA	c.2237_2251del15	1	−	−	−
p. E746-T751del insVA	c.2237_2251 > TGG	1	−	−	−
p.delL747-A750insP	c.2238_2248 > GC	1	−	−	−
p. E747-A751del	c.2240_2254del15	1	−	−	−
p. L747-S752del insS	c.2240_2257del18	1	−	−	−
p.G719C	c.2155G > T	1	−	−	−
p.T790M	c.2369C > T	2	−	−	−
p.L858R	c.2573T > G	14	−	−	−
p.L861Q	c.2582T > A	3	−	−	−
p.L861R	c.2582T > G	1	−	−	−
***PIK3CA***		**7**	**58**	**1**	**50**
p.E542K	c.1624G > A	2	10	1	8
p.E545A	c.1634A > C	−	1	−	−
p.E545G	c.1634A > G	−	1	−	1
p.E545D	c.1635G > T	−	1	−	−
p.E545K	c.1633G > A	3	21	−	10
p.E545Q	c.1633G > C	−	2	−	−
p.Q546K	c.1636C > A	−	1	−	2
p.Q546E	c.1636C > G	−	−	−	1
p.Q546P	c.1637A > C	−	1	−	−
p.Q546R	c.1637A > G	−	4	−	−
p.M1043I	c.3129G > A	−	1	−	−
p.H1047L	c.3140A > T	−	3	−	6
p.H1047R	c.3140A > G	2	11	−	22
p.G1049R	c.3145G > C	−	1	−	−

In total, we detected 24 *KRAS*, 9 *NRAS*, 4 *BRAF*, 12 *EGFR*, and 14 *PIK3CA* different mutations. Notably, among the 24 *KRAS* different mutations, we reported the occurrence of novel complex mutation in two distinct samples of colon adenocarcinoma: it is a dinucleotide mutation c.33_34TG > CT p.G12C (FIG. 4A), not yet listed in the COSMIC database (COSMIC Release v73).

In our heterogeneous series, mutations most commonly occurred in the *KRAS* oncogene (362/605, 60%), followed by *PIK3CA* (116/605, 19%), *BRAF* (58/605, 10%), *EGFR* (41/604, 7%), and *NRAS* (28/605, 5%). Mutation occurrences in most genes had strong tendency toward mutual exclusivity, except for *PIK3CA* in colon, lung and pancreatic cancers that, on the opposite, tended toward co-occurrence: out of 67 *PIK3CA* mutations, 47 were co-occurring mutations (70%). In particular, 45 adenocarcinomas (41 colon, 2 lung, 2 pancreas) exhibited co-occurring *KRAS* and *PIK3CA* mutations; two colon adenocarcinomas had concomitant *BRAF* and *PIK3CA* mutations. A melanoma case had *NRAS* mutation co-occurring with *PIK3CA* mutation. The *PIK3CA* tendency toward co-occurrence was not observed in breast carcinomas: out of 47 *PIK3CA* mutations, only one case showed the presence of concomitant *KRAS* and *PIK3CA* mutations.

In colorectal carcinomas, 1 case presented concomitant *KRAS* and *NRAS* mutations and 2 cases had concomitant G12D and G13D *KRAS* mutations.

Overall, *KRAS* mutations were found in 195/386 colorectal cancers (50%), 108/316 lung cancers (34%), 52/65 pancreatic cancers (80%), 3/11 biliary tract cancers (27%), and 2/180 (1%) breast cancers; *BRAF* mutations were found in 30/62 melanomas (48%), 22/386 colorectal cancers (6%), 5/316 lung cancers (2%), and 1/65 pancreatic cancer (1%); *NRAS* mutations were found in 14/62 melanomas (23%), and 14/386 colorectal cancers (4%); *PIK3CA* mutations were found in 61/406 colorectal cancers (15%), 47/180 breast cancers (26%), 7/316 lung cancers (2%), 2/65 pancreatic cancer (3%), 1/62 melanomas (2%), and 1/11 biliary tract cancers (9%); *EGFR* mutations were found in 41/316 lung cancers (13%).

## DISCUSSION

In this study we report the rapid development of a high throughput, cost-effective and simple strategy for the detection of clinically relevant mutations in a variety of solid tumors such as carcinomas of the lung, colon, breast, pancreas, biliary tract, and melanomas.

Given the increasingly critical role of molecular investigations in the management of cancer patients, there is an immediate need for robust, high-quality diagnostic tests. The complexities of NGS technologies and data analysis are slowing their wide spread availability in the molecular pathology laboratories, which must readily comply with regulatory, quality and professional standards [19–21]. While working to optimize and validate this revolutionary approach, diagnostics laboratories need a robust genotyping platform which can carry-on their daily routine testing.

The presented approach uses the MALDI-TOF mass spectrometry technology, associated with iPLEX assays, that allows analysis of multiple mutations in a single investigation [22]. This technology utilizes small (80 base pairs) PCR products which are optimal for amplification of FFPE DNA templates.

The PentaPanel platform assesses single nucleotide polymorphisms in 56 hotspots of the 5 most clinically relevant cancer genes, *KRAS, NRAS, BRAF, EGFR* and *PIK3CA* for a total of 221 detectable mutations. Indeed, the PentaPanel genotyping approach covers point mutations of the mandatory biomarkers for non-small cell lung carcinomas, colorectal carcinomas, melanomas, and satisfactorily meets the needs of a high quality throughput pathology laboratory, such as accuracy, sensitivity, hands-on time, and costs. This is a cost effective method, especially when complex testing of numerous mutations is requested: for example the *RAS*-testing (*KRAS* and *NRAS* mutations in exon 2, 3 and 4) in colorectal cancer performed by PentaPanel produced a significant reagent cost saving of 30% over real-time PCR or pyrosequencing analysis. Moreover, the high levels of multiplexing and automation of PentaPanel genotyping reduce personnel costs below those of other standard methods.

*EGFR* exon 19 deletions were not included in this platform and they were detected by sizing electrophoresis. Efficient approaches for point mutations analysis are generally not optimal for insertions/deletions [23]. In fact, investigating the entire mutation spectrum of deletions within the *EGFR* gene is feasible by this method but both a high number of assays and an increased amount of DNA sample would be required for the test.

The presented results highlight the PentaPanel advantages: it was successfully used to interrogate different DNAs isolated from routinely processed specimens (FFPE, frozen, and cytological samples); it required small amount (as low as 8ng) of DNA template for the assessment of multiple genes status simultaneously; it could genotype 10 patients in the same run with a practical turnaround time of 2 days, including data analysis and evaluation; and its hand-on timing easily allowed more than a run a day.

Importantly, the PentaPanel platform analyzed 24 highly frequently mutated sites bidirectionally, meaning that it comprised two separate assays assessing the presence of the mutation in both forward and reverse strands in separate wells. This is crucial for the detection of complex mutations such as dinucleotide substitutions occurring in both *BRAF* and *KRAS* genes.

Particularly, in melanomas the *BRAF* complex mutations were 20% of the total *BRAF* detected mutations (6/30: 1 p.V600E2 and 5 pV600K), revealing that such events are not rare and they must be taken into account when performing *BRAF* testing. The correct *BRAF* status identification is crucial since evidence of clinical benefit to vemurafenib treatment is accumulating for patients with mutations other than V600E [24]. In fact, the spectrum of tested mutations is another critical issue successfully addressed by the PentaPanel compared to the other targeted methods (real-time PCR-based methods), currently used for routine diagnostics. The real-time PCR assays are rapid and highly sensitive [15] but they often require a rather significant amount of DNA, not always available in limited biopsy/cytologic samples, and more importantly, underestimate not pre-designed mutations. Therefore, these tests might not cover all clinically relevant mutations [25–27], consequently excluding patients from the potential benefits of targeted therapy.

The PentaPanel bidirectionally strategy also allows to confirm each result at a specific site by two assays in different wells in the same run and no additional validation steps are needed. Notably, this approach markedly reduces the possibility of both false-negative and false-positive results: the presented data showed a concordance of 100% between PentaPanel and the Sanger sequencing integrated with the more sensitive LNA-PCR/Sequencing results. Indeed, we found that the PentaPanel achieves a specificity of 100% and no false positive mutation calls were observed in our series; the analytical sensitivity of detecting mutant alleles was as low as 5%.

The application of the PentaPanel to routine molecular analysis of 1025 samples showed a genotyping high success rate (99,5%), with a 2% rescue of successfully analyzed samples by this approach compared to Sanger sequencing.

In conclusion, this well established and robust high throughput technology was overall easy to be set up and rapidly introduced in the routine molecular diagnostics of our Institution; importantly the presented data support its valid clinical application, and compliance to molecular testing guidelines.

Although the mass spectrometry based genotyping will not be a definitive cancer diagnostics platform as being able to detect only targeted mutations, the PentaPanel can provide the immediate and accurate multiplex approach for clinically relevant gene mutation analysis in many solid tumors; moreover, its extreme flexibility and limited costs can allow both rapid implementation and application to newly identified biomarkers for target therapies selection, and its effectiveness across many diseases can be particularly relevant in multiple clinical trials, including the new basket trial approach, aiming to identify appropriate targeted drug combination strategies.

## MATERIALS AND METHODS

### Tumor samples

We retrospectively collected anonymized solid tumor samples from 1025 consecutive patients that had undergone routine diagnostic somatic mutation analysis at the San Raffaele Hospital Pathology Unit (Milan, Italy) between September 2013 and December 2014.

A comprehensive written informed consent was signed for the procedures (fine needle aspiration, biopsies and surgical resections) that produced the tissue samples and the diagnostic workup. All information regarding the human material was managed using anonymous numerical codes. Clinical data and follow up information were not used for this study. According to our country's legislation, since it was a retrospective study, with no direct patient involvement, the ethical approval and patients consent for the study were not required (Official Gazette No. 301 of December 30, 2014).

All samples were handled in compliance with the Helsinki declaration.

The study included 386 colon adenocarcinomas, 316 lung adenocarcinomas, 62 melanomas and 256 other malignancies (180 breast carcinomas, 65 pancreatic and 11 biliary tract adenocarcinomas). 910 samples derived from formalin-fixed, paraffin-embedded (FFPE) specimens, 73 were obtained from fresh frozen tissues (both biopsies or surgical specimens) and 42 were cytological stained smears.

All the diagnosis were confirmed by two pathologists (GA and DC) and tumor-rich areas (>50%) were selected in order to perform manual macrodissection prior DNA extraction. Coverslips were removed from stained cytological samples using xylene followed by hydration and air-drying.

For tissue blocks, both FFPE and frozen OCT-embedded, a variable number (5–10) of 5 μm unstained sections were prepared, depending on the tumor size.

For all samples total genomic DNA was isolated by automated extraction using the Magcore Nucleic Acid Extractor (RBC Bioscience, Taiwan) following the manufacturer's protocols. Quality and quantity of isolated DNA was assessed by a NanoDrop 2000c spectrophotometer (Thermo Fisher Scientific, Waltham, MA, USA).

### Mutation analysis using sequenom massARRAY genotyping assays

Briefly, the Sequenom MassARRAY approach included designing multiplexed specific assays that use primers flanking the mutation site and extension primers that bind adjacent to the mutation site. After the amplification of the region of interest, a primer extension reaction was carried out. The extension reaction included sequence-specific hybridization and sequence-dependent single base termination (iPLEX) that generated different products for the mutated and wild type alleles, each with its unique mass, then identified using mass spectrometry.

Assay amplification primers and extension oligos were designed using MassARRAY Assay Design software v. 4.0 (Sequenom, USA) with a maximum of 12 multiplexed assays per well. In particular, 8-well multiplexed assays were designed to assess single nucleotide polymorphisms (SNPs) involving 56 hotspots of the *KRAS, NRAS, BRAF, EGFR* and *PIK3CA* genes (PentaPanel). For 24 sites of *EGFR, KRAS, NRAS*, and *BRAF*, which are mutated at high frequency as reported by the Catalogue Of Somatic Mutations In Cancer (COSMIC), we included both duplicate amplifications, in separate wells, and bidirectional single base pair extension (Figure 1); the PentaPanel comprised a total of 80 assays, 48 of them present in both forward and reverse direction, covering 221 mutations in the 5 genes (Figure 2).

Amplification, nucleotide dephosphorylation and single base primer extension by i-PLEX^®^ Gold chemistry were performed according to the manufacturer's protocol (Sequenom). A MALDI-TOF mass spectrometer (MassARRAY Compact, Sequenom) was used to resolve extension products; data analysis was performed utilizing MassARRAY Typer Analyzer software (Sequenom).

Dilution series of FFPE-derived DNA with known mutations in a background of wild type FFPE-derived DNA (20%, 10%, 5%, 2.5% mutant to wild type DNA ratios) together with commercially available pre-designed reference standards (Horizon Diagnostics, UK) were used to detect the allelic analytical sensitivity of the assays.

The PentaPanel platform was then used to analyze the *KRAS, NRAS, BRAF, EGFR* and *PIK3CA* genes status in the previously described series of 1025 cases, utilizing 40ng of genomic DNA template for all the samples. Using a 96-well plate it was possible to investigate 10 cases in the same run, including both positive and negative controls, with a turnaround time of 2 days.

SPSS version 17.0 was used for statistical analysis.

### Mutation analysis by standard Sanger sequencing

We analyzed the entire coding region of exon 2, exon 3, and exon 4 of both *KRAS* and *NRAS*, exon 15 of *BRAF*, exon 18, exon 20, exon 21 of *EGFR*, exon 9 and exon 20 of *PIK3CA*. PCR primers sequences and thermal conditions are summarized in supplemental data (Supplementary Table 1).

Amplified products were purified using MinElute PCR Purification Kit (Qiagen Gmbh, Germany) and sequenced in both directions using the BigDye Terminator v1.1 Cycle Sequencing Kit (Applied Biosystems, USA), according to the manufacturer's protocol, on an ABI Prism 3130 Genetic Analyze running ABI Prism DNA Sequence Analysis Software. To increase the sensitivity of standard Sanger sequencing we modified the standard PCR sequencing assay by the addition of 20 pmol of Locked Nucleid Acid (LNA) probe (Exiqon, Denmark) complementary to the wild type sequence of the *KRAS* (codons 12–13) and *BRAF* (codons 598–601), as previously described [28].

### Length analysis of fluorescently labelled PCR products for EGFR deletions in exon 19

Deletions in exon 19 of *EGFR* gene were determined by fragment length analysis after PCR amplification with the use of FAM-labeled primer as previously described [3]. Separation was done with a four-color laser-induced fluorescence capillary electrophoresis system (ABI Prism 3130 Genetic Analyzer, Applied Biosystems). The collected data were evaluated with the Gene Scan Analysis Software. All mutant were confirmed by DNA direct sequencing.

## SUPPLEMENTARY TABLE


