# Supplementary material for: Rapid targeted somatic mutation analysis of solid tumors in routine clinical diagnostics
Source: Oncotarget. 2015 Sep 2;6(31):30592–603. doi: 10.18632/oncotarget.5190 (PMC4741554; doi:10.18632/oncotarget.5190)
Supplement: Supplementary file 1 [file oncotarget-06-30592-s001.pdf]

SUPPLEMENTARY TABLE

Supplementary Table S1: *BRAF, KRAS, NRAS, EGFR* and *PIK3CA* primer sequences and PCR reaction conditions.

GENE	PRIMER SEQUENCE	PCR PROGRAM
KRAS EXON 2<br><br><br>KRAS EXON 3<br><br><br>KRAS EXON 4	FOR – GGTGGAGTATTTGATAGTGTATTAACC<br>REV – TCATGAAAATGGTCAGAGAAACC<br><br>FOR – TGCACTGTAATAATCCAGACTGTG<br>REV – TGCATGGCATTAGCAAAGAC<br><br>FOR – GATATTTGTGTTACTAATGACTGTGCT<br>REV – CCTCTCAAGAGACAAAAACATTTAC	96°C 2 min<br>45 cycles 96°C 30 sec<br>58°C 30 sec<br>72°C 30 sec<br>72°C 7 min
NRAS EXON 2<br><br><br>NRAS EXON 3<br><br><br>NRAS EXON 4	FOR – AGAACCAAATGGAAGGTCACA<br>REV – CCGACAAGTGAGAGAGACAGGA<br><br>FOR – TAGCATTGCATTCCCTGTGG<br>REV – CAGAGAAAATAATGCTCCTAGTACCTG<br><br>FOR – GCGAGTAAAAGACTCGGATGA<br>REV – TGAATATGGATCACATCTCTACCA	96°C 2 min<br>45 cycles 96°C 30 sec<br>58°C 30 sec<br>72°C 30 sec<br>72°C 7 min
BRAF EXON 15	FOR – TGCTTGCTCTGATAGGAAAATG<br>REV – TGATTTTTGTGAATACTGGGAAC	96°C 2 min<br>45 cycles 96°C 30 sec<br>58°C 30 sec<br>72°C 30 sec<br>72°C 7 min
EGFR EXON 18<br><br><br>EGFR EXON 20<br><br><br>EGFR EXON 21	FOR – AGGGCTGAGGTGACCCTTGT<br>REV – TCCCCACCAGACCATGAGAG<br><br>FOR – ATCGCATTCATGCGTCTTCA<br>REV – ATCCCCATGGCAAACTCTTG<br><br>FOR – GCTCAGAGCCTGGCATGAA<br>REV – CATCCTCCCCTGCATGTGT	96°C 2 min<br>10 cycles 96°C 30 sec<br>65°C–55°C* 30 sec<br>72°C 30 sec<br>35 cycles 96°C 30 sec<br>58°C 30 sec<br>72°C 30 sec<br>72°C 7 min
PIK3CA EXON 9<br><br><br>PIK3CA EXON 20	FOR – AAATTTATTGAAAATGTATTTGCTTTTTC<br>REV – TCCATTTTAGCACTTACCTGTGACTC<br><br>FOR – ATGCCAGAACTACAATCTTTTGATGAC<br>REV – GCTTTCATTTTCTCAGTTATCTTTTCAGT	96°C 2 min<br>45 cycles 96°C 30 sec<br>55°C 30 sec<br>72°C 30 sec<br>72°C 7 min

*Touchdown: −1°C each cycle
